# New concept of myocardial longitudinal strain reserve assessed by a dipyridamole infusion using 2D-strain echocardiography: the impact of diabetes and age, and the prognostic value

**DOI:** 10.1186/1475-2840-12-84

**Published:** 2013-06-07

**Authors:** Thomas Cognet, Paul-Louis Vervueren, Laurent Dercle, Delphine Bastié, Rainui Richaud, Matthieu Berry, Pauline Marchal, Matthieu Gautier, Audrey Fouilloux, Michel Galinier, Didier Carrié, Pierre Massabuau, Isabelle Berry, Olivier Lairez

**Affiliations:** 1 Department of Nuclear Medicine, University Hospital of Rangueil, Toulouse, France; 2 Department of Cardiology, University Hospital of Rangueil, Toulouse, France; 3 Cardiac Imaging Centre, University Hospital of Rangueil, Toulouse, France; 4 Laboratory of Epidemiology, CJF-INSERM, 94-06, Toulouse, France; 5 Medical School of Rangueil, University Paul Sabatier, Toulouse, France; 6 Medical School of Purpan, University Paul Sabatier, Toulouse, France; 7 Department of Cardiology, Toulouse University Hospital, 1, avenue Jean Poulhès, TSA 50032, 31059, Toulouse Cedex 9, France

**Keywords:** Dipyridamole, Speckle-tracking echocardiography, Diabetes, Strain reserve

## Abstract

**Aims:**

Although dipyridamole is a widely used pharmacological stress agent, the direct effects on myocardium are not entirely known. Diabetic cardiomyopathy can be investigated by 2D-strain echocardiography. The aim of this study was to assess myocardial functional reserve after dipyridamole infusion using speckle-tracking echocardiography.

**Methods:**

Seventy-five patients referred for dipyridamole stress myocardial perfusion gated SPECT (MPGS) were examined by echocardiography to assess a new concept of longitudinal strain reserve (LSR) and longitudinal strain rate reserve (LSRR) respectively defined by the differences of global longitudinal strain (GLS) and longitudinal strain rate between peak stress after dipyridamole and rest. Twelve patients with myocardial ischemia were excluded on the basis of MPGS as gold standard.

**Results:**

Mean LSR was −2.28±2.19% and was more important in the 28 (44%) diabetic patients (−3.27±1.93%; p = 0.001). After multivariate analyses, only diabetes improved LSR (p = 0.011) after dipyridamole infusion and was not associated with glycaemic control (p = 0.21), insulin therapy (p = 0.46) or duration of the disease (p = 0.80). Conversely, age (p = 0.002) remained associated with a decrease in LSR. LSSR was also correlated to age (p = 0.005). Patients with a LSR < 0% have a better survival after 15 months (log-rank p = 0.0012).

**Conclusion:**

LSR explored by 2D speckle-tracking echocardiography after dipyridamole infusion is a simple and new concept that provides new insights into the impact of diabetes and age on the myocardium with a potential prognostic value.

## Introduction

Dipyridamole is a widely used pharmacological agent to test coronary reserve in patients referred for stress myocardial perfusion imaging. The mechanism of stress with dipyridamole implies a coronary vasodilatation, which leads to the detection of myocardial ischemia through coronary steal phenomena. Dipyridamole mainly increases the flow supply in the subendocardial layer by decreasing vascular resistance [1], but the resulting effects on myocardium and especially on myocardial strain have not been explored yet. The myocardial functional reserve between peak stress and rest reflects the ability of the myocardium to improve its function during stress testing. Stress testing is recommended in order to detect myocardial ischemia [2], but the role of the myocardial functional reserve during stress remains undervalued even if it provides important information in some situations such as hypertrophic cardiomyopathy or hypothyroidism [3,4]. At the same time, the ability of two-dimensional (2D) strain echocardiography to precisely assess myocardial function provides new possibilities for an accurate measurement of the myocardial function reserve in stress conditions [5,6] and especially in diabetes in which longitudinal strain is altered at baseline [7]. Actually, diabetic patients have early myocardial damage at baseline that requires to be detected by means of modern tools such as biomarkers and imaging [8].

The aim of this study was to assess the effects of dipyridamole on the myocardium in a population of patients referred for myocardial perfusion imaging by gated single-photon emission computed tomography (MPGS) and to explore the myocardial functional reserve with dipyridamole by means of a longitudinal strain reserve with speckle-tracking echocardiography.

## Methods

### Study population

Patients referred for MPGS with previous or non-coronary artery disease (CAD) and without history of myocardial infarction were prospectively included from March to September 2011. All patients underwent a complete physical exam before inclusion. Exclusion criteria were documented allergies to dipyridamole, asthma, systolic blood pressure < 90 mmHg, decompensated heart failure or acute angina, significant valvular disease, hypertrophic cardiomyopathy, atrial fibrillation and the possibility to induce stress by effort. All patients were informed of the study design and agreed to the protocol before inclusion. Patients with identified MPGS ischemia were also excluded of the analysis.

### Dipyridamole testing and MPGS protocols

All enrolled patients underwent MPGS with intravenous dipyridamole pharmacologic stress using the standardized protocols from the European Association of Nuclear Medicine / European Society of Cardiology guidelines [9]. Caffeinated beverages, foods and medications, and medications containing methylxanthine were avoided for at least 12 hours prior to stress testing. Dipyridamole was given as a continuous infusion intravenously at 0.6 mg/kg over the course of 4 minutes. Arterial pressure was recorded before infusion, every 2 minutes during stress and also at the peak of dipyridamole effects (8 minutes). Stress MPGS was performed for all patients with a weight-adjusted dose of 300–400 MBq of 99mTc-tetrofosmin injected 3 minutes after the completion of the dipyridamole infusion. MPGS was acquired 15 to 30 minutes after a radiotracer injection using a Symbia T6 (Siemens Healthcare, Erlangen, Germany) double-headed gamma camera equipped with low-energy, high-resolution collimators. Data was acquired for 180° with 64 frames of 30 and 20 second durations at stress and at rest, respectively; a 64 × 64 matrix; 8-frame gating; and a 20% window centred on the 140-keV photo peak of Tc-99m. Rest MPGS was performed only if stress MPGS was considered as pathological, with a 2-fold-higher dose of 99mTc-tetrofosmin injected at least 3 hours after the stress testing. All patients underwent low-dose CT using a Symbia T6 system (Siemens Healthcare, Erlangen, Germany) for attenuation correction (130 keV, 30 to 45 mAs).

### Echocardiographic protocol

Echocardiography for all patients was performed at rest and 3 minutes after the completion of the dipyridamole infusion, i.e. at peak of stress, with a Imagic KM 60 (Kontron Medical, Saint-Germain en Laye, France) using a 2.5 MHz transducer. A complete two-dimensional grey scale echocardiography including the three standard apical views (four, three and two chambers) with a frame rate > 75 frame/s was performed for each patient. Left ventricle ejection fraction (LVEF) and volumes were assessed before and after the dipyridamole infusion as diastolic parameters. Myocardial strain was measured using speckle-tracking echocardiography.

### Data analysis and interpretation

The 17-segments model, as defined by the American Society of Echocardiography, was used to examine both echocardiography and MPGS [10]. The apex segment was then excluded for the analysis.

**Figure 1:**
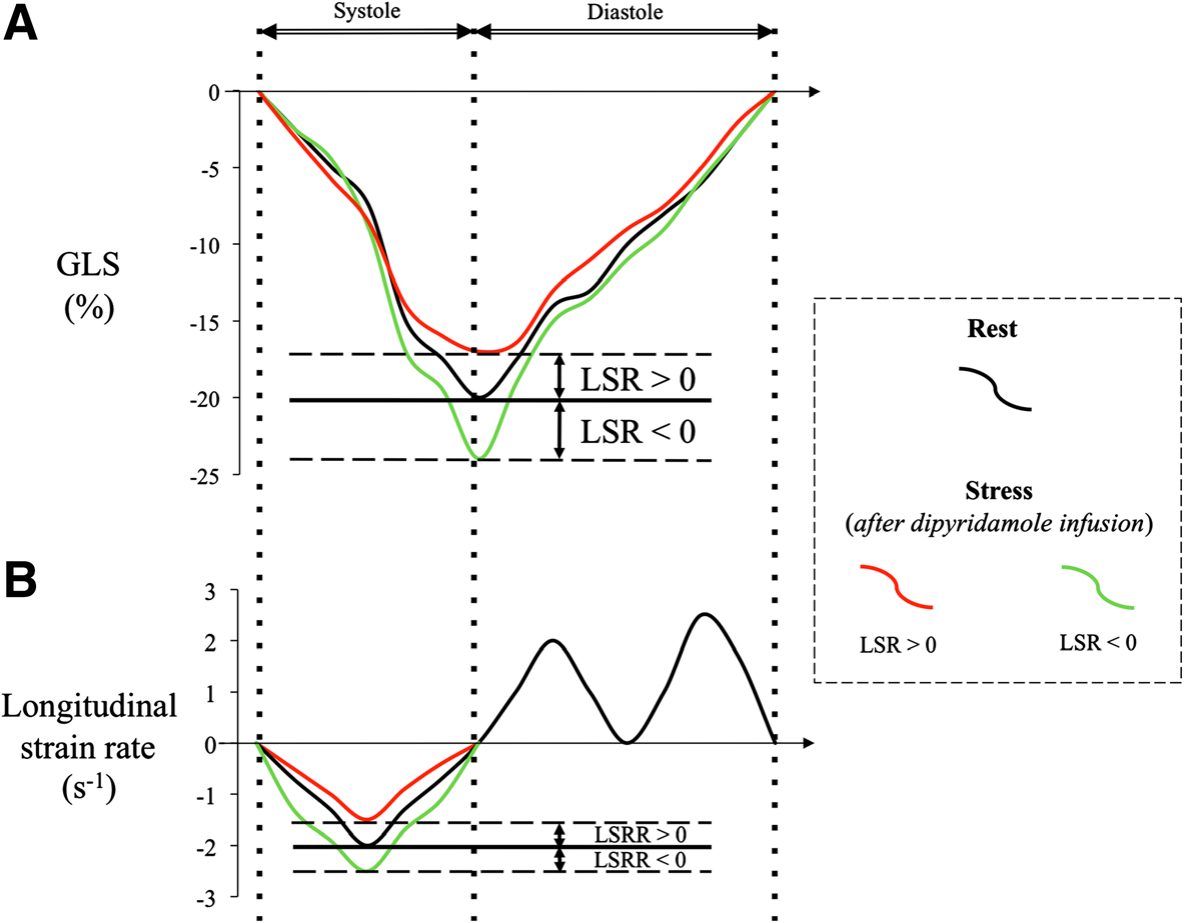
**Schematic representation of global longitudinal strain (GLS) curves (Panel A) and longitudinal strain rate curves (Panel B) focused of the systolic phase of the cardiac cycle.** Improvement of longitudinal strain reserve (negative LSR) and longitudinal strain rate reserve (negative LSRR) in green curves and decrease of LSR and LSRR (positive LSR and LSRR) in red curves both after dipyridamole infusion as compared to baseline values (black curves).

For the echocardiography, digital data of 3 consecutive heart cycles were recorded and transferred to a personal computer with My Lab Desk workstation (Kontron Medical, Saint-Germain en Laye, France) for offline analysis. The endocardial border was defined manually in end systole and automatically tracked frame by frame. Operator assessed optimal evaluation of both quality of tracking and region of interest. Global longitudinal strain (GLS) was obtained by averaging all segmental longitudinal strain curves computed from the conventional apical two-, three- and four-chamber views. Longitudinal strain reserve (LSR) was defined by the difference between peak systolic global longitudinal strain at the peak of vasodilatation with dipyridamole and at rest. The longitudinal strain rate was determined for the left ventricle as the maximal strain rate value (calculated as the temporal derivative of strain) during the ejection phase. The longitudinal strain rate reserve (LSRR) was similarly obtained (Figure 1). Left ventricular ejection fraction (LVEF) was assessed by transthoracic echocardiography using the conventional apical two- and four-chamber views and the modified Simpson’s method.

For MPGS studies, off-line analysis was performed on Syngo MI Applications software (Siemens Healthcare, Erlangen, Germany). The images were assessed visually and by applying automated methods. A single blinded observer interpreted echocardiographic and MPGS studies. Myocardial ischemia was defined by at least one reversible myocardial perfusion defect between stress and rest myocardial perfusion gated-SPECT and was expressed by the number of segments affected.

### Follow up

Data about the occurrence of adverse events were obtained from medical records by direct patients’ interviews or from the referring physician. The primary end point was defined by all-cause mortality. Patients unable to be interviewed up to 6 months at the date of follow-up were considered as lost to follow-up.

### Statistical analysis

Data were expressed as mean +/− SD. Nominal values were expressed as numbers and percentages. Normality was tested by the Kolmogorov-Smirnov test. The association between the mean values of continuous normally distributed variables were compared using unpaired and paired Student’s t test and the Mann–Whitney rank sum test was used when the samples were not normally distributed or had unequal variances. Comparison between multiple groups was performed with a variance analysis (ANOVA). Nominal variables were investigated by the χ^2^ test. Linear regression analysis was used to investigate the relation between LSR-LSRR and variables. Conventional variables correlated with LSR with a p value < 0.05 at first univariate analyses were used to build the final multivariate stepwise model. Receiver operating characteristic (ROC) curves were computed to determine optimal cut-off point for longitudinal strain reserve as well as to calculate area under the curve (AUC) to determine prognostic significance. Multivariate Cox regression model was built to identify echocardiographic parameters associated with all-cause mortality. Survival curve was determined according to the Kaplan-Meier method, and cumulative event rates compared by means of the log-rank test. Differences were considered statistically significant for p-values of < 0.05. All analyses were performed on SPSS software version 20 (SPSS Inc., Chicago, Illinois).

## Results

### Population

**Figure 2:**
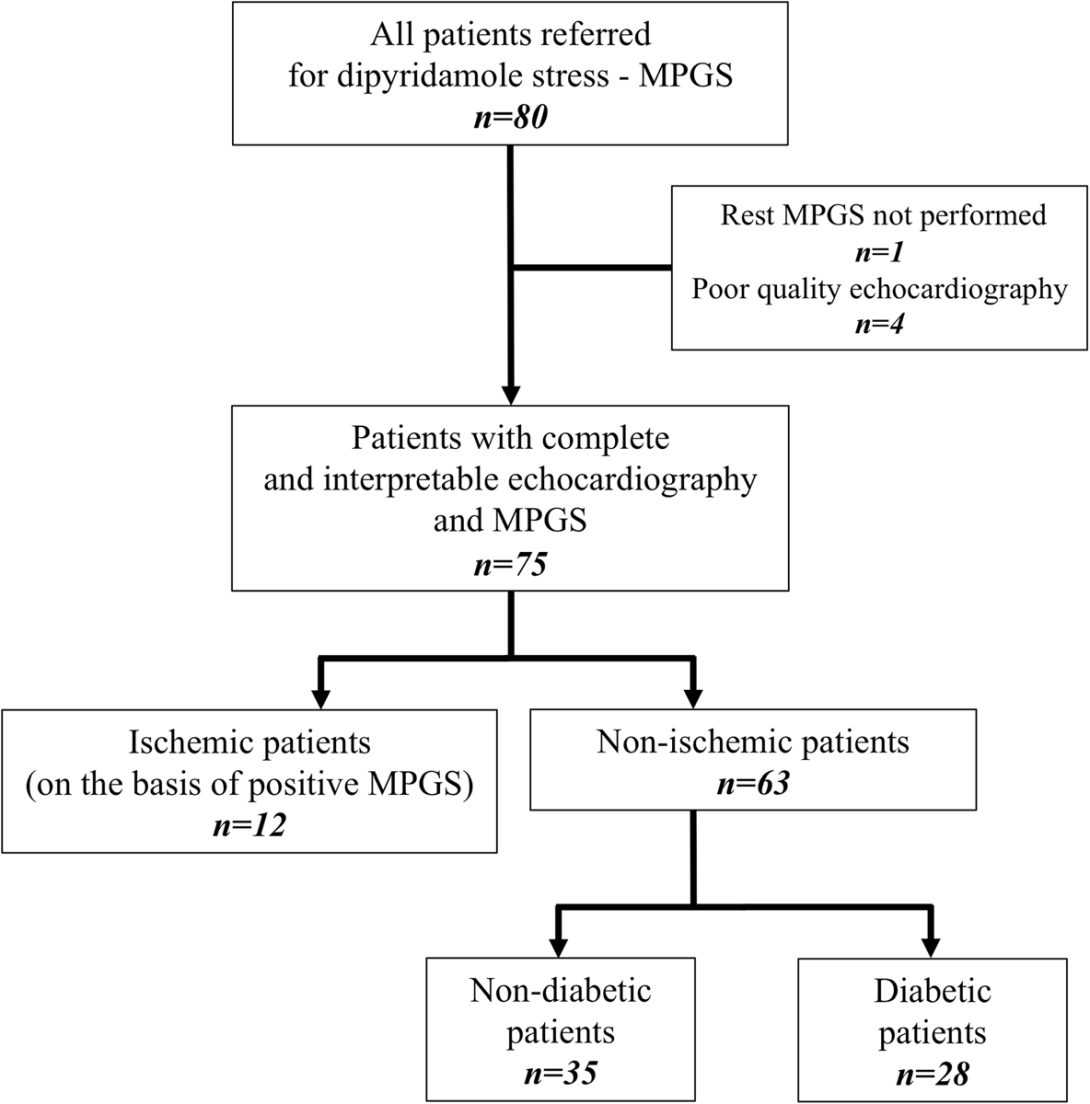
**Flow chart of the study population.** MPGS = Myocardial perfusion imaging by gated single-photon emission computed tomography.

Eighty patients were prospectively included. Four patients (5%) were excluded from the analysis due to a poor resolution of 2D echocardiography as a consequence of poor ultrasonic window (obesity or pulmonary disease) that did not allow speckle-tracking imaging and 1 (<1%) due to refusal of gated-SPECT after echocardiography. Among the 75 other patients included, 12 were excluded for MPGS ischemia with at least one or more reversible defect segment (Figure 2). Male represented 59% of the 63 patients finally enrolled in the study. The mean age was 70 ± 11 years with a median age of 71 ranging from 46 to 90 years old. Twenty-six patients (41%) had a previous history of coronary artery disease and 28 (44%) suffered from diabetes. Diabetes lasted less than five years in 10/28 patients. Thirty-nine patients (62%) reported a NYHA stage 2 and the mean LVEF was 51±14%. Baseline characteristics are presented in Table 1.

**Table 1:** Baseline characteristics

**Variable**	**All patients (n=63)**	**Diabetic (n=28)**	**Non-diabetic (n=35)**	***p-value***
Age (yrs)	70 ±11	70±10	72±10	**0.04**
Male (%)	37 (59)	18 (64)	19 (54)	0.42
BMI (kg/m^2^)	26.3 ± 3.9	28.1±4.0	24.8±3.2	**0.001**
SBP (mmHg)	137 ± 21	135±16	138±24	0.55
DBP (mmHg)	76±11	75±12	77±11	0.64
HR (beats/min)	70 ± 14	69±12	71±16	0.59
**Dyspnea status (%)**				
NYHA I	12 (19)	7 (25)	5 (14)	0.83
NYHA II	39 (62)	16 (57)	23 (66)	0.65
NYHA III	12 (19)	5 (18)	7 (20)	0.72
**Coronary risk factors (%)**				
Hypertension	45 (71)	19 (48)	26 (71)	0.58
Current smoking	27 (43)	12 (43)	15 (43)	0.99
Hypercholesterolemia	32 (51)	14 (50)	18 (51)	0.91
Family history of cardiovascular	4 (6)	2 (7)	2 (5)	0.82
disease				
**Previous coronary artery disease (%)**	26 (41)	11 (39)	15 (43)	0.78
**Medications (%)**				
ACE inhibitors/ARB	38 (62)	16 (57)	22 (63)	0.44
Beta-blockers	30 (48)	12 (43)	18 (51)	0.50
Calcium antagonists	25 (40)	10 (36)	15 (43)	0.56
**Diabetic characteristics**				
Duration of diabetes (yrs)	-	12.0±8.8	-	-
HbA1c (%)	-	7.0±2.4	-	-
Insulin therapy (%)	-	20 (71)	-	-
Oral therapy (%)		12 (43)		
*Metformin therapy (%)*	-	8 (29)	-	-
Retinopathy (%)	-	11 (39)	-	-
Peripheral arterial disease (%)	-	15 (54)	-	-
*Supra aortic*	-	6 (21)	-	-
*Lower limb*	-	11 (39)	-	-

Values are presented as n (%) or mean +/− SD.

*BMI* = body mass index; *SBP* = systolic blood pressure; *DBP* = diastolic blood pressure; *HR* = heart rate; *ACE* = angiotensin-converting enzyme; *ARB* = angiotensin receptor blocker; *LVEF* = left ventricle ejection fraction; *LVEDV and LVESV* = left ventricle end diastolic and end systolic volume.

### Blood pressure and heart rate during stress

Hemodynamic during dipyridamole infusions and echocardiographic examinations remained unchanged for all patients. The decrease of systolic blood pressure with dipyridamole after stress testing was not only insignificant for the whole population (136±22 at rest vs. 130±21 mmHg after dipyridamole infusion, p = 0.14) but the diabetic patients in comparison to the non-diabetics also showed a non-significant variation of systolic blood pressure (−3.8±12.4 vs. -2.9±31.6 mmHg; p = 0.88, respectively). Results are similar regarding the diastolic blood pressure (p = 0.09). The mean heart rate increased from 70±14 beats/min at rest to 80±18 beats/min after the dipyridamole infusion (p < 0.001) showing the pharmacological effect of dipyridamole. No examination had to be stopped for safety reasons.

### Effects of dipyridamole on strain reserve

The effects of dipyridamole on LSR according to baseline characteristics, coronary risk factors, dyspnea and medications are presented in Table 2. In our general population, the mean GLS before dipyridamole infusion was −14.5±4.2% and reached −16.8±4.5% at the maximum effect of vasodilatation. Consequently, the mean LSR was −2.28±2.19%. LSR did not depend on systolic blood pressure (p = 0.99), diastolic blood pressure (p = 0.57) or heart rate (p = 0.85) changes during stress, as LSRR with p-values of 0.89, 0.57 and 0.17, respectively for systolic blood pressure, diastolic blood pressure and heart rate.

**Table 2:** Effects of dipyridamole on longitudinal strain reserve

	**Coef. 95% for LSR**	
		**p-value**
**Baseline characteristics**		
Age (yrs) *	−0.49	**<0.0001**
Male **†**	−2.45	0.46
Female	−2.04	
BMI (kg/m2) *	0.25	**0.045**
**Dyspnea status †**		
NYHA I	−2.33	0.98
NYHA II	−2.30	
NYHA III	−2.17	
**Coronary risk factors †**		
Hypertension	−2.33	0.77
No hypertension	−2.15	
Diabetic	−3.27	**0.001**
Non-diabetic	−1.49	
Current smoking	−2.92	**0.045**
No current smoking	−1.80	
Hypercholesterolemia	−2.29	0.98
No hypercholesterolemia	−2.27	
Family history of cardiovascular disease	−0.90	0.19
No family history of cardiovascular disease	−2.38	
**Previous coronary artery disease (CAD) †**		
CAD	−2.02	0.43
No CAD	−2.46	
**Medications †**		
ACE inhibitors/ARB	−2.50	0.44
No ACE inhibitors/ARB	−2.07	
Beta-blockers	−1.80	0.10
No beta-blockers	−2.72	
Calcium antagonists	−2.00	0.41
No calcium antagonists	−2.47	

*LSR* = longitudinal strain reserve; *BMI* = body mass index; *ACE* = angiotensin-converting enzyme; *ARB* = angiotensin receptor blocker.

*: Spearman correlation.

†: ANOVA.

**Figure 3:**
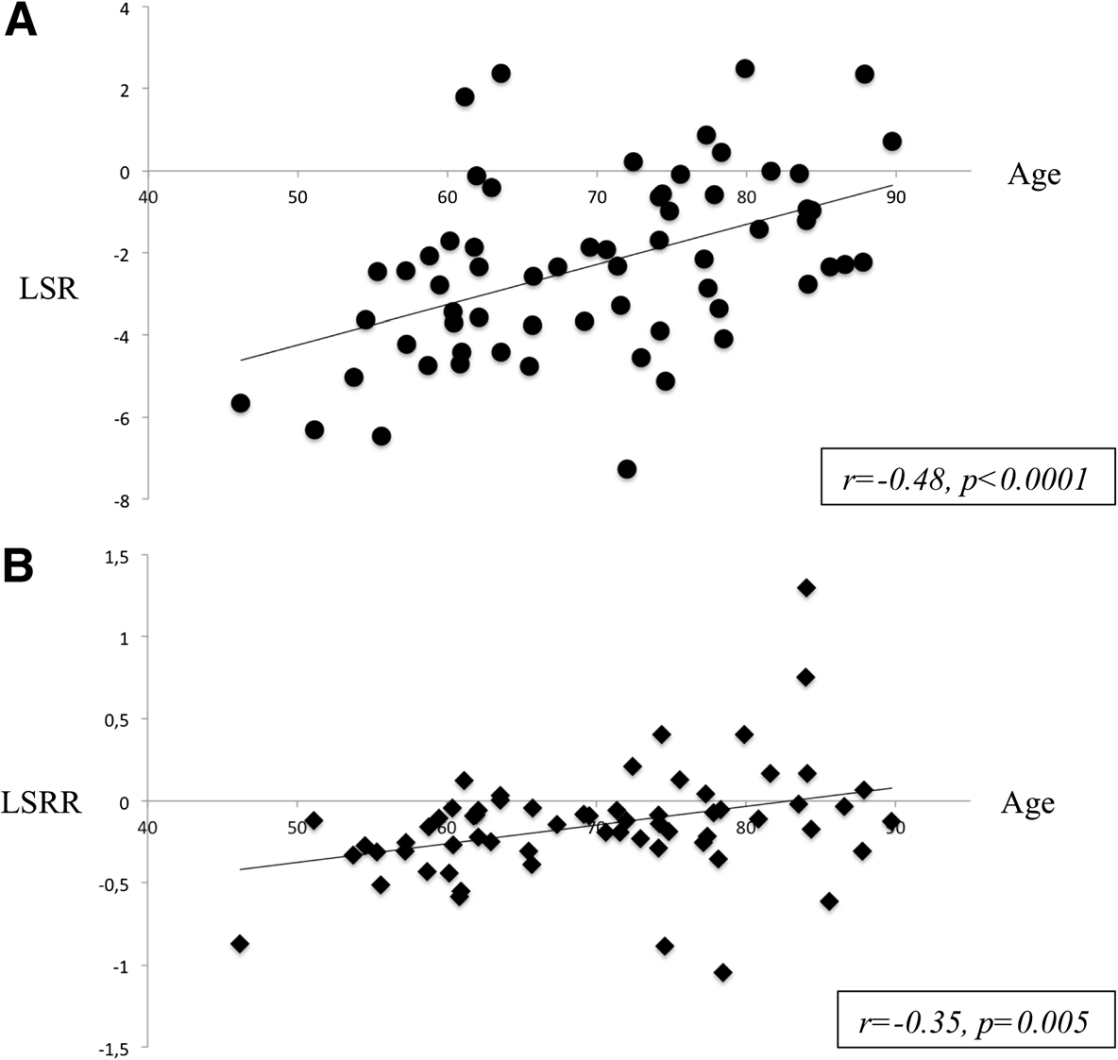
**Impact of age on LSR and LSRR A.** Correlation between increasing age and decreased longitudinal strain rate reserve in Panel **B**. LSR = longitudinal strain reserve, LSRR = longitudinal strain rate reserve.

By univariate analysis, only age was associated with a decrease of LSR after dipyridamole infusion whereas patients with diabetes, higher Body Mass Index (BMI) and current smoking showed an improvement of LSR (Table 3). Increasing age was significantly correlated to a decrease of LSR (p < 0.0001) as presented in Figure 3. As shown in Table 4, no difference was observed between diabetic and non-diabetic patients for GLS before stress (−13.9±3.7 vs. -15.0±4.5%; p = 0.30) and after the dipyridamole infusion (−17.2±4.2 vs. -16.5±4.8%; p = 0.55) but LSR was higher in the diabetic population (−3.27±1.93 vs. -1.49±2.08%; p = 0.001). Moreover, GLS of diabetic increased significantly by 24% in stress conditions (p = 0.003). Among the 28 patients with diabetes, 22 of them presented also overweight, defined as a BMI > 25 kg/m2 (p < 0.004). GLS before dipyridamole infusion was not different between patients with or without overweight (−14.2±3.6 vs. -15.0±4.9%; p = 0.43) but LSR was significantly higher in patients with overweight (−2.83±1.85 vs. -1.50±2.42%; p = 0.016). After a multivariate analysis, only age (p = 0.001) remained independently associated with a decrease of LSR after the dipyridamole infusion. Conversely, LSR remained significantly improved only in diabetic patients (p = 0.008). Among all echocardiographic parameters at baseline and after stress, including systolic, diastolic, hemodynamic and speckle-tracking parameters, only LSR was modified according to the diabetic status (Table 4). LSR was not correlated to the duration of diabetes (p = 0.80) or HbA1c level (p = 0.21) and was not influenced by dedicated treatments especially insulin therapy (p = 0.46), the presence of retinopathy (p = 0.43) or peripheral vascular disease (p = 0.34).

**Table 3:** Univariate and multivariate linear regression model for longitudinal strain reserve

	**Multivariate analysis**			
	**Coef. [95% CI]**	**p-value**	**Coef. [95% CI]**	**p-value**
Age	**−0.10 [−0.14 – -0.05]**	**<0.0001**	**−0.08 [−0.13 – -0.04]**	**0.001**
Gender (Male)	0.42 [−0.71 – 1.54]	0.46		
BMI	**0.14 [0.01 – 0.28]**	**0.045**		
Hypertension	0.18 [−1.05 – 1.41]	0,77		
Diabetic	**1.79 [0.77 – 2.81]**	**0.001**	**1.33 [0.36 – 2.30]**	**0.008**
Current smoking	**1.11 [0.02 – 2.20]**	**0.045**		
Hypercholesterolemia	0.02 [−1.09 – 1.13]	0.98		
Family history of cardiovascular disease	−1.48 [−3.72 – 0.77]	0.19		
ACE inhibitors/ARB	0.44 [−0.70 – 1.57]	0.44		
Beta-blockers	−0.92 [−2.01 – 0.17]	0.10		
Calcium antagonists	−0.47 [−1.60 – 0.66]	0.41		
Previous CAD	−0.44 [−1.57 – 0.68]	0.43		

*BMI* = body mass index; *ACE* = angiotensin-converting enzyme; *ARB* = angiotensin receptor blocker; *MPGS* = myocardial perfusion gated SPECT; *CAD* = coronary artery disease.

**Table 4:** Echocardiographic parameters according to diabetic status

**Echocardiographic parameters**	**All patients (n=63)**	**Diabetic (n=28)**	**Non-diabetic (n=35)**	***p-value***
**Rest**				
LVEF (%)	50.7±14.4	49.8±13.0	51.4±15.5	*0.66*
EDV (ml)	103.7±46.9	106.7±46.6	101.2±47.6	*0.65*
ESV (ml)	53.7±39.4	56.6±36.7	51.3±41.8	*0.60*
TVI LVOT (cm)	22.7±6.0	19.3±5.0	19.3±5.5	*0.96*
GLS (%)	−14.5±4.2	−13.9±3.7	−15.0±4.5	*0.30*
Longitudinal Strain rate (s^-1^)	−1.19±0.35	−1.15±0.26	−1.22±0.40	*0.43*
E (cm.s^-1^)	77±25	78±26	75±25	*0.60*
A (cm.s^-1^)	81±29	79±28	82±29	*0.77*
E/A (cm.s^-1^)	1.07±0.58	1.14±0.57	1.01±0.59	*0.41*
DT (s)	205±86	197±88	210±85	*0.56*
**Stress**				
LVEF (%)	54.6±13.9	54.3±14.0	54.9±13.9	*0.89*
EDV (ml)	98.8±41.6	98.8±36.3	98.9±45.9	*0.99*
ESV (ml)	48.8±35.6	48.6±31.6	48.9±38.9	*0.97*
TVI LVOT (cm)	22.7±6.0	23.0±5.0	22.5±6.8	*0.76*
GLS (%)	−16.8±4.5	−17.2±4.2	−16.5±4.8	*0.55*
Longitudinal Strain rate (s^-1^)	−1.34±0.44	−1.33±0.52	−1.35±0.37	*0.86*
E (cm.s^-1^)	84±23	83±23	85±23	*0.75*
A (cm.s^-1^)	89±33	88±32	89±35	*0.85*
E/A (cm.s^-1^)	1.13±0.77	1.15±0.61	1.10±0.89	*0.83*
DT (s)	179±73	177±76	180±71	*0.87*
∆ **(stress – rest)**				
∆ LVEF (%)	3.9±8.4	4.6±9.8	3.5±7.2	*0.60*
∆ EDV (ml)	−4.8±25.3	−7.9±30.0	−2.3±20.9	*0.39*
∆ ESV (ml)	−4.9±20.5	−8.0±24.1	−2.4±17.0	*0.28*
∆ TVI LVOT (cm)	3.4±2.5	3.7±2.6	3.2±2.3	*0.39*
LSR (%)	−2.28±2.19	−3.27±1.93	−1.49±2.08	***0.001***
LSRR (s^-1^)	−0.15±0.34	−0.17±0.43	−0.12±0.26	*0.57*

*LVEF* = left ventricle ejection fraction; *EDV* = end diastolic volume; *ESV* = end systolic volume, *TVI LVOT* = time-velocity integral in the left ventricular outflow tract, *GLS* = global longitudinal strain; *DT* = mitral deceleration time, *LSR* = longitudinal strain reserve; *LSRR* = longitudinal strain rate reserve.

LSRR was only associated with aging (p = 0.005) and was not influenced by diabetes (p = 0.57). Moreover, longitudinal strain rate increased by 16% between baseline and peak stress in the diabetic population (p = 0.11).

### Prognosis and follow-up

**Figure 4:**
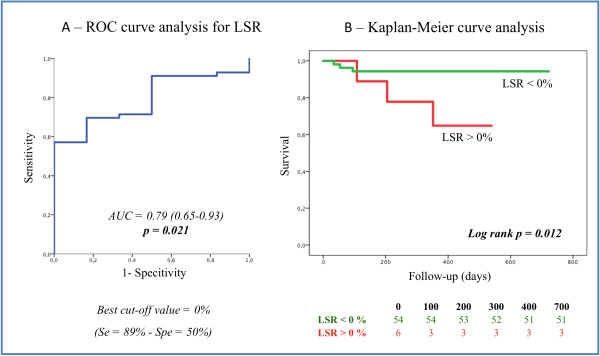
**Prognostic value of LSR A.** Kaplan-Meier curve representing the impact of a positive LSR on all-cause mortality in Panel **B**. LSR = longitudinal strain reserve. AUC = area under the curve, Se = sensitivity and Spe = specitivity.

During a mean follow-up of 15±5 months, 6 (10%) patients reached the primary endpoint. Only one patient was lost to follow-up and was excluded for survival analysis. After multivariate analysis, only the left ventricle end systolic volume at stress (HR: 1.047 [95% C.I: 1.018 – 1.075]; p = 0.001; Table 5), the difference of left ventricle end systolic volumes between stress and rest (HR: 1.065 [95% C.I: 1.019 – 1.113]; p = 0.005) and a positive LSR (HR: 15.493 [95% C.I: 1.419 – 169.182]; p = 0.025) remained independently associated with all-cause mortality. ROC curve analysis in Figure 4 identified positive LSR (cut-off value of 0%) as a predictor of all-cause mortality with a sensitivity of 89% and a specificity of 50%, for an area under the curve of AUC = 0.79 (p = 0.021). The Kaplan-Meier analysis showed better survival in patients with a negative LSR (log-rank p = 0.012). All cause-mortality in the diabetic population was associated with a lower LSR (−0.59±1.17 vs. -3.60±1.77%; p = 0.028) but prognosis was not significantly better in the diabetic population compared to the non-diabetic since they have a better LSR (p = 0.102 vs. p = 0.446).

**Table 5:** Echocardiographic parameters associated with all-cause mortality in a multivariate Cox regression model

	**Univariate analysis**		**Multivariate analysis**	
	**HR [95% CI]**	***p-value***	**HR [95% CI]**	***p-value***
**Rest**				
LVEF (%)	0.889 [0.821 - 0.964]	***0.004***		
EDV (ml)	1.019 [1.007 - 1.031]	***0.002***		
ESV (ml)	1.020 [1.007 - 1.033]	***0.002***		
GLS (%)	1.335 [1.091 – 1.633]	***0.005***		
**Stress**				
LVEF (%)	0.898 [0.839 - 0.960]	***0.002***		
EDV (ml)	1.026 [1.012 - 1.040]	***<0.0001***		
ESV (ml)	1.034 [1.017 - 1.051]	***<0.0001***	1.047 [1.018 - 1.075]	***0.001***
GLS (%)	1.481 [1.155 - 1.899]	***0.002***		
∆ **(stress – rest)**				
∆ LVEF (%)	0.962 [0.874 - 1.059]	*0.430*		
∆ EDV (ml)	1.003 [0.970 - 1.037]	*0.862*		
∆ ESV (ml)	1.044 [1.006 - 1.084]	***0.022***	1.065 [1.019 - 1.113]	***0.005***
LSR (%)	1.395 [0.973 – 2.001]	*0.070*		
LSR > 0	6.012 [1.211 - 29.856]	***0.028***	15.493 [1.419 - 169.182]	***0.025***

*LVEF* = left ventricle ejection fraction; *EDV* = end diastolic volume; *ESV* = end systolic volume, *TVI LVOT* = time-velocity integral in the left ventricular outflow tract, *GLS* = global longitudinal strain; *LSR* = longitudinal strain reserve; *LSRR* = longitudinal strain rate reserve.

## Discussion

LSR assessed with dipyridamole, defined by the difference between GLS after and before a dipyridamole infusion, is a new concept of myocardial functional reserve. Our study shows a LSR increase in patients with diabetes that decreases with aging with a potential interest in prognosis evaluation.

2D-strain echocardiography with speckle-tracking imaging enables a general analysis of the left ventricle at rest or during stress [11]. Regional analysis during MPGS and perfusion echocardiography with dipyridamole are complementary for the assessment of CAD [12] but for the first time, we are reporting the effects of dipyridamole on global longitudinal strain by means of 2D-speckle tracking echocardiography. This study highlights a new concept of LSR during stress with dipyridamole. Palmieri and al. previously experienced a myocardial reserve by means of Doppler tissue imaging. Despite different pharmacological effects, low doses of dobutamine in patients with type 1 diabetes have similar effects, compared to dipyridamole in our study, by improving both global longitudinal strain and longitudinal strain rate of at least 29% [13].

Different deformation modalities such as longitudinal strain [14,15] or torsion [16-18] can be modified by left ventricular load but even if dipyridamole has several systemic effects that can lead to hemodynamic changes [19], we show that there are no blood pressure impacts or heart rate variations on LSR.

After a multivariate analysis, only aging is associated with a decrease of LSR during stress with dipyridamole. This result is consistent with the consequences of aging on myocardial deformation at rest: global longitudinal strain declines at rest with aging in a healthy population [20], especially in basal segments [21]. Consistent results were also found with longitudinal strain rate in baseline [22]. These results could be partly explained by a reduced coronary flow reserve with aging [23] as described with myocardial ischemia [24]. Therefore, LSR may reflect the physiological age of the myocardium.

Conversely, diabetes is associated with an increase in LSR whereas GLS before the dipyridamole infusion in the diabetic patients is lower but not statistically different than non-diabetic patients. These results at baseline are different from the studies of Ernande et al. in which GLS was impaired at rest in patients with diabetes [25], even sometimes before diastolic dysfunction [26]. This difference for GLS at rest could be explained by the recent description of impaired coronary microvascular function in type 2 diabetic patients without CAD [27]. Our smaller population of diabetic patients and a population partly composed of patients with previous CAD might explain this difference. Several studies confirm the endothelial dysfunction secondary to diabetes [28] but we show, for the first time, the mechanic consequences of this endothelial dysfunction in diabetic patients, which lead to an exacerbated response to arterial vasodilatation induced by dipyridamole. These results are consistent with the hemodynamic findings of Picchi and al. who previously described an increased basal coronary blood flow at rest in diabetes as a cause of decreased coronary flow reserve with adenosine. Interestingly, as we described with GLS, coronary blood flow is not altered after adenosine in the diabetic group compared to the non-diabetic group [29]. The lack of any decrease in systolic or diastolic blood pressure in the diabetic group might also explain these results. Actually, reduced myocardial perfusion with vasodilatator in diabetic patient is associated with a significant decrease in blood pressure, a consequence of autonomic neuropathy [30]. Coronary metabolic vasodilatation mainly depends on both nitric oxide metabolism, which is impaired in diabetic patients [31], and on adenosine pathways. The predominant vasodilatation effects of dipyridamole through A_2A_-adenosine receptors, which are increased in the hearts of diabetic rats, [32] could explain the increase of LSR in the diabetic population. LSR is not influenced by duration of diabetes in contrast to baseline [33]. Glycaemic control, treatments and vascular disease other than CAD do not influence either LSR. In parallel, left ventricular diastolic function is impaired precociously in patients with insulin resistance and glucose metabolism disorders even without overt diabetes [34] but the diastolic functional reserve defined by Jellis et al. is not altered by diabetes during effort conditions [35]. Diabetes seems to alter first the contractile function as described with dobutamine stress echocardiography [36] while myocardial perfusion seems to be maintained [37]. This hypothesis may explain the improvement of the LSR in diabetic patients. As a result, LSR appears to be an interesting and sensitive tool to explore the impact of diabetes on myocardium non-invasively, regardless of the characteristics of the diabetes.

Strain rate is load dependent and reflects the regional difference in contractility [38]. In our study, LSRR is not influenced by hemodynamic changes induced by dipyridamole. Therefore, LSRR reflects changes in contractility due to myocardial injury only. Moreover, in diabetes, the stability of longitudinal strain rate with stress and the insignificant increase in LSRR between diabetic and non-diabetic patients results in a homogeneous and stable regional contractility. This may be the consequence of an overall and homogeneous myocardial injury. Because subendocardial longitudinal fibres are the most vulnerable in pathological conditions, we deliberately focused our analysis on longitudinal deformation [39]. Longitudinal strain is the most reliable and studied parameter of deformation modalities and the comparison of radial strain results in diabetic populations is not reliable among different studies [25,40].

A positive LSR that reflects lack of myocardial functional reserve after dipyridamole infusion appears promising to predict all-cause mortality in a population of patients referred for MPGS even when ischemia is excluded. The cut-off value of 0% of LSR is easy to measure and allows therefore a rapid and reliable evaluation in routine clinical practice.

However, our study has several limitations. First, presence of autonomic neuropathy [41] but also abdominal visceral adipose tissue [42], and the evaluation of aortic stiffness [43] or oxidative stress [44], all associated with myocardial function impairment, could have provided important information. In parallel, our population of diabetic patients is to small to define subset groups according to glycaemic control that could influence evolution of LSR and LSRR during follow up. Unfortunately, ischemia cannot be ruled out with certainty and therefore might interfere with our results. MPGS is an efficient and validated exam to assess ischemia and CAD and even if patients with at least only one reversible defect segment were excluded, pluritroncular patients could lead to false negative tests. Moreover, coronary angiography should have been interesting to differentiate respective impacts of epicardial coronary artery disease and microvascular dysfunction on strain reserve among patients with diabetes.

Finally, further prospective studies are necessary to define the interaction between diabetes and LSR and a potential prognostic value in diabetic patients. The use of the selective adenosine A_2A_ receptor agonist vasodilator stress agent could be interesting in this context [45]. However, the present results suggest that addition of LSR to dipyridamole stress myocardial contrast perfusion echocardiography could improve its prognostic value [46].

## Conclusion

LSR assessed after a dipyridamole infusion is a new concept of stress examination using speckle-tracking imaging. LSR increases in the diabetic population and warrants special attention for examinations in the elderly population. Myocardial function reserve assessed by LSR after dipyridamole with speckle-tracking echocardiography may be of interest for the evaluation of both prognosis and impact of co-morbidities.

## Consent

Written informed consent was obtained from the patient for publication of this report and any accompanying images.

## Abbreviations

2D: Two-dimensional; MPGS: Myocardial perfusion imaging by gated single-photon emission computed tomography; CAD: Coronary artery disease; GLS: Global longitudinal strain; LSR: Longitudinal strain reserve; LSRR: Longitudinal strain rate reserve; LVEF: Left ventricle ejection fraction; AUC: Area under the curve; BMI: Body mass index.

## Competing interests

Doctor Lairez received research support (equipment and software) from Kontron for speckle-tracking echocardiography. However, the current study was not supported by those grants.

## Authors’ contributions

TC researched and analyzed data and wrote the manuscript. PLV performed the statistical analysis. LD recorded follow-up information. DB, RR, MB, PM, MG, AF researched data. MG, DC, PM, IB contributed to discussion and reviewing. O.L. led the study, revised the manuscript and gave final approval of the version to be published. All authors read and approved the final manuscript.
